# Factors Influencing the Choice of Radiology Subspecialty Among Radiology Trainees in Sudan

**DOI:** 10.7759/cureus.32555

**Published:** 2022-12-15

**Authors:** Salma Abdelrahman, Rufaida Mohamed, Alsafa Mostafa, Dana Eltyeb, Eman Mohamedalamin, Ibrahim Elkhidir, Wadah Mohamed, Nafisa Medani, Balgees Ibrahim

**Affiliations:** 1 General Surgery, Ibrahim Malik Teaching Hospital, Khartoum, SDN; 2 Intensive Care Unit, Diabetes and Endocrine Hospital, Khartoum, SDN; 3 Accident and Emergency, Diabetes and Endocrine Hospital, Khartoum, SDN; 4 Internal Medicine, Haj El-Safi Teaching Hospital, Khartoum, SDN; 5 Faculty of Medicine, University of Khartoum, Khartoum, SDN; 6 General and Colorectal Surgery, Northern Care Alliance, Manchester, GBR

**Keywords:** sudan medical specialization board, factors, subspecialty, radiology, residency, training

## Abstract

Background and objective

There are numerous reasons why radiologists would be interested in seeking additional fellowship training, some of which are personal, such as the possibility of bettering their career prospects, while others are work-related. This study aimed to identify whether the Sudanese radiology trainees wanted to pursue fellowship and what were the motivating and restricting factors affecting their career choices.

Methods

This was a re-do research of a study from Saudi Arabia previously published in the Cureus journal in 2019. This was a descriptive cross-sectional study conducted among the radiology registrars training under Sudan Medical Specialization Board (SMSB) in 2022 (n=90). By using convenient sampling, 74 of the 90 registrars were contacted, and a response rate of 81% (n=60) was achieved. Data were collected using a pre-tested self-administered online questionnaire. Data were analyzed using IBM SPSS® Statistics version 25.0 (IBM Corp., Armonk, NY). A p-value ≤0.05 was considered statistically significant.

Results

The majority of the trainees in our study were females (61.7%, 37/60). More than 93% (n=56) of our participants were training in Khartoum, the capital of Sudan. The most commonly chosen subspecialties in our study were as follows: neuroradiology (33.3%, n=20) body imaging (26.7%, n=16), and interventional radiology (25%, n=15). In contrast, nuclear medicine (1.7%, n=1) and emergency radiology (3.3%, n=2) were among the least popular subspecialties. The top influencing factors among our trainees in choosing a subspecialty included "strong personal interest," "lifestyle," and "area of strong personal knowledge." The most common factors preventing trainees from opting for a fellowship were "financial restriction" (55%, n=33) and "family obligation" (28.3%, n=17). Of those with no plans to subspecialize, 75% (six out of eight) stated that the lack of a fellowship program in Sudan is a possible deterrent. A statistically significant association was found between gender and the choice of subspecialty in interventional radiology and women’s/mammogram imaging. Our findings revealed that there are currently no trainees in the first year of radiology residency because the last selection exam had been conducted in 2019. Despite the current unavailability of subspecialty training in Sudan, 75% (n=45) of trainees in our study were interested in joining a local program for fellowship training in the future.

Conclusion

Radiology trainees in Sudan share similar interests and influencing and restricting factors when pursuing subspecialty training, as reported in the literature. Unlike other countries, females predominate in the field of radiology training in Sudan at the moment. Radiologists from Sudan who are interested in subspecializing usually travel abroad for training; and once they find better prospects, many of them may not return. Programs offering subspecialization locally could mitigate the attrition of radiologists in Sudan. When designing subspecialty training programs in Sudan, stakeholders should use knowledge of influential factors and understanding of subspecialty decision trends among radiology trainees as a reference point. To the best of our knowledge, this study is the first of its kind to be conducted in this field in Sudan.

## Introduction

Subspecialization involves devoting intellectual resources toward detailed learning about a narrower and specific area of a specialty [1]. The radiology subspecialty must comprise a distinct area of knowledge that cannot be incorporated into the general radiology curriculum. In addition, it must have unique applicability backed up by evidence of improved patient care that a subspecialist's skillset can offer, and structured formal training with apparent benefits that do not negatively impact the existing general radiology training or other radiology subspecialties [2]. Many factors that drive the interest of a radiologist in pursuing further fellowship training have been identified in the literature, some of which are personal, such as the chance of improving career prospects, while others are job-related [3-8].

Africa suffers from a chronic lack of access to radiology training programs, both at the level of the diagnostic radiology (DR) residency and the radiology specialty (RS) fellowship. It has been shown that only 18 of the 54 sovereign African nations (excluding disputed territories) report having well-established DR residency programs, and only eight of those nations report having any subspecialty programs, with information being readily available for only five of them: Egypt, Ethiopia, Kenya, South Africa, and Tanzania [2]. In Africa, radiology postgraduate residency and subspecialization appear to have lagged behind the rest of the world [2].

Radiology training was started in Sudan in 1992 at the University of Khartoum. However, it was only in 2003 that the program came under the regulatory authority of the Sudan Medical Specialization Board (SMSB), the body responsible for medical specialty training in Sudan [9]. Sudan, a developing African nation, faces numerous obstacles to successfully adopting radiology subspecialization. These challenges include a lack of financial resources and knowledge, inadequate infrastructure and equipment, politics, and the emigration of radiologists to foreign countries [2]. Only four vascular interventional radiologists (VIR) are currently employed in Sudan (all of them practicing in the country's capital, Khartoum), which illustrates the current state of radiology fellowship in the country. All interventionists in Sudan have received their training in either Jordan or Malaysia since an interventional radiology training fellowship is not available in the country [9]. There is scarce data on radiologists in Sudan. Currently, the SMSB does not offer a subspecialty training program.

This paper aims to better understand the factors that influence the career choices of radiology trainees in Sudan. To the best of our knowledge, this is the first study of its kind in this field in Sudan. Understanding the trends among radiology trainees in choosing a subspecialty and gaining knowledge about the factors that influence them might help and guide decision-makers in establishing subspecialty training programs in the country.

## Materials and methods

This was a re-do research of a study from Saudi Arabia previously published in the Cureus journal in 2019 [10]. It was a descriptive cross-sectional study involving registrars in the radiology training program under SMSB. The radiology training program is four-year long in Sudan; there are currently three batches of trainees, from year two to four, with 90 registrars in total. The sample size was calculated using the Cochran formula with a confidence interval (CI) of 95%, a margin of error of 5%, and a population proportion of 50% variability; the sample size was calculated to be 74. The chosen trainees were contacted through Google Forms in November 2022, and the response rate was 81% (n=60). Data were collected using a pre-tested, self-administered online questionnaire adopted from the previous study [10]. The questionnaire included questions about demographic data such as age, gender, current year of training, and working area, as well as questions about preferred subspecialties and factors preventing and influencing fellowship selection (11 questions in total). Data were refined and analyzed using IBM SPSS® Statistics version 25.0 (IBM Corp., Armonk, NY). Significant differences in the demographic characteristics in a specific subspecialty compared to all other subspecialties (combined) were evaluated using the Chi-square or Fisher's exact test, as appropriate. A p-value ≤0.05 was considered statistically significant.

Ethical clearance was obtained from the Ethics Board Committee of the Ministry of Health. Informed written consent was taken from each participant. The research purpose was explained in the header of the questionnaire, and the participants were informed that they could withdraw at any time. No details that can identify the participants were collected. The questionnaire was anonymous, with no identifying data for the researchers. Each participant was assigned a unique number only for analysis purposes.

## Results

Out of the total 74 radiology trainees contacted, 60 participated in this study, showing a response rate of 81%; 37 of the participants were females (61.7%) (Table 1). As mentioned previously, there were no first-year trainees (R1) in the country at the time of the study; there were 32, 34, and 24 trainees in R2, R3, and R4, respectively. All three years were almost equally represented in the study, as follows: 33.3% (n=20), 35.0% (n=21), and 31.7% (n=19), respectively (Table 1).

**Table 1:** Demographic data and willingness to pursue fellowship training

Variables	Frequency	Percent
Gender		
Female	37	61.70%
Male	23	38.30%
Total	60	100.00%
What is your current year of training?		
R2	20	33.30%
R3	21	35.00%
R4	19	31.70%
Total	60	100.00%
Do you plan to pursue fellowship training?		
Doubtful	11	18.30%
No	8	13.30%
Yes	41	68.30%
Total	60	100.00%

The majority of the trainees are currently training in Khartoum state (93.4%, n=56), while three trainees are in North Kordofan state (4.9%), and one trainee is in Al-Jazirah state (1.6%). When asked about their willingness to pursue a fellowship after residency, 41 participants (68.3%) expressed their desire to subspecialize in the future; on the other hand, 11 were doubtful (18.3%), and only eight did not want to go for a fellowship (13.3%) (Table 1). Of the latter, six participants (75%) stated that the lack of a fellowship program in Sudan is a possible deterrent.

Of those who planned to pursue a fellowship in the future (n=52), 39 (75%) had decided their area of interest before residency, and only 13 (25%) decided during or after residency. Of the total participants, 34 (56.7%) reported that they were interested in pursuing research after the fellowship, and 12 (20%) were doubtful. However, 23.3% of the participants had no plans for fellowship or no interest in research (n=14).

**Figure 1:**
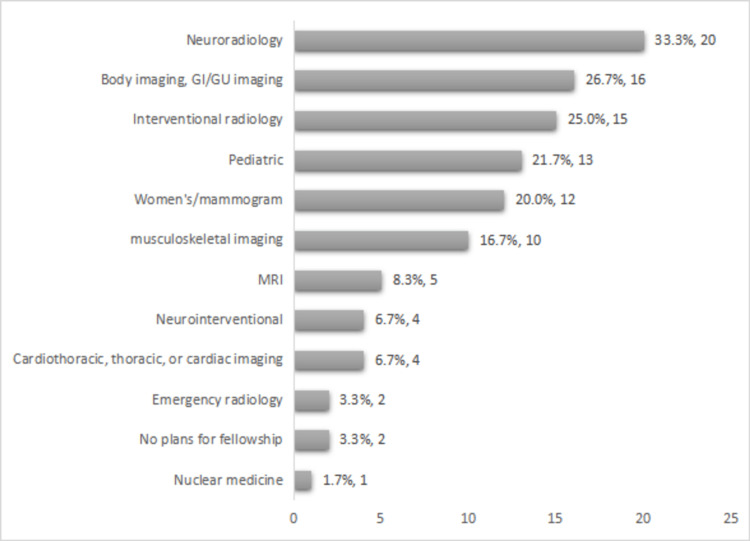
Choice of subspecialty among trainees

Regarding the areas of interest in the fellowship, the participants had the choice to select more than one specialty; neuroradiology was chosen by 20 trainees (33.3%). Body imaging, interventional radiology, pediatric, and women's/mammogram imaging were preferred by 16 (26.7%), 15 (25%), 13 (21.7%), and 12 (20%) participants, respectively. MRI; cardiothoracic, thoracic, or cardiac imaging; and neurointerventional were selected by 8.3% (n=5), 6.7% (n=4), and 6.7% (n=4), respectively. Nuclear medicine was chosen by a single participant (1.7%), while emergency radiology was selected by two participants (3.3%) (Figure 1).

**Figure 2:**
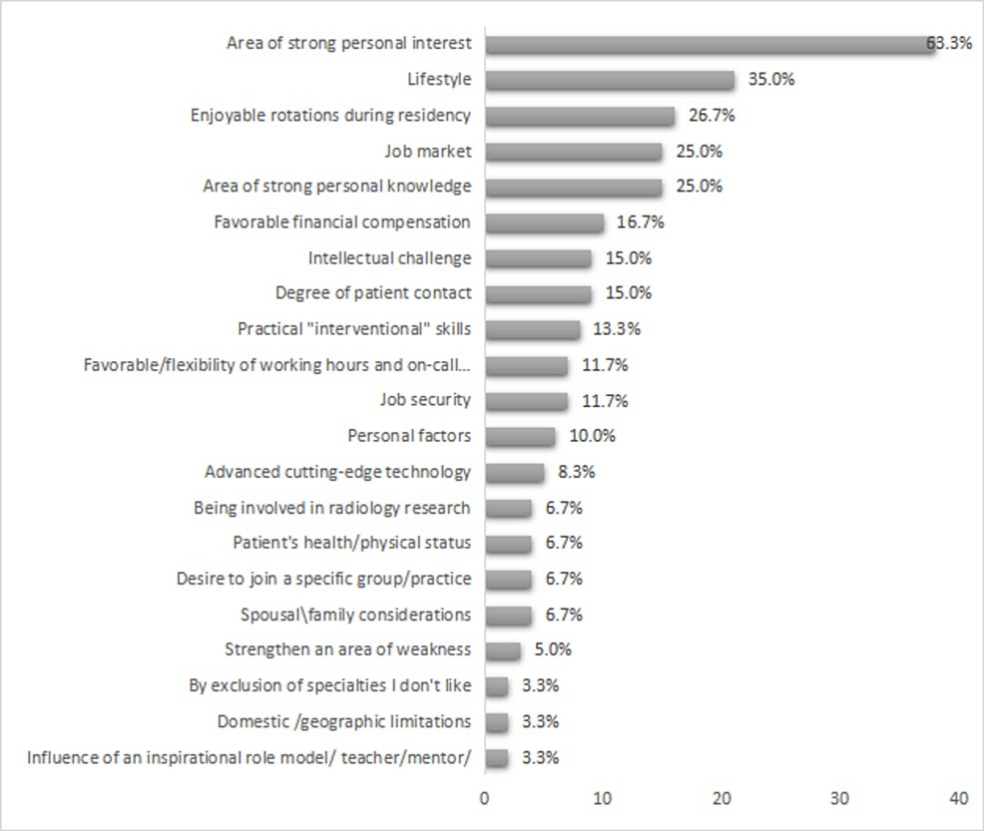
Factors affecting the choice of subspecialty

As for the factors affecting the subspecialty choice, "being an area of strong personal interest" was chosen by 38 trainees (63.3%); "lifestyle," "being an area of strong personal knowledge," "enjoyable rotations during residency," and "Job market" were selected by 21 (35.0%), 16 (26.7%), 15 (25.0%), and 15 (25.0%) trainees, respectively. Other determinants of subspecialty choice were as follows: "favorable financial compensation" (16.7%, n=10), "degree of patient contact" (15.0%. n=9), "intellectual challenge" (15.0%, n=9), "practical interventional skills" (13.3%, n=8), "job security" (11.7%, n=7), "favorable/flexibility of working hours and on-call commitments" (11.7%, n=7), "personal factors" (10.0%, n=6), and "advanced cutting-edge technology" (8.3%, n=5). "By exclusion of specialties I don't like," "domestic/geographic limitations," and "to strengthen an area of weakness," were selected by only two participants each (3.3%) (Figure 2).

**Figure 3:**
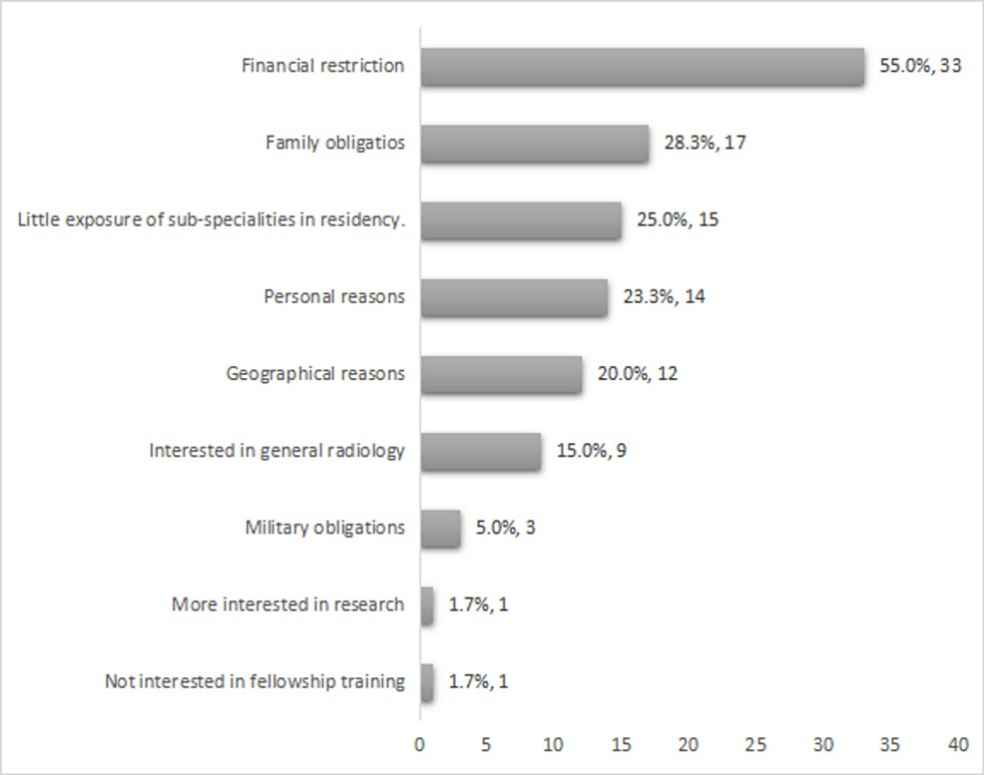
Factors restricting the pursuit of subspecialty

Regarding the factors preventing radiologists from choosing the desired subspecialty, 33 trainees (55.0%) reported "financial restriction" being a limitation. Others mentioned "family obligations" (28.3%, n=17), "little exposure to subspecialties during residency" (25%, n=15), or "personal reasons" (23.3%, n=14); 20% of the trainees (n=12) claimed that "geographical reasons" had limited their choice of subspecialty. Other limitations that the participants reported were "military obligations" (5%, n=3), "having no desire for fellowship training" (1.7%, n=1), or "having more interest in research" (1.7%, n=1) (Figure 3).

**Figure 4:**
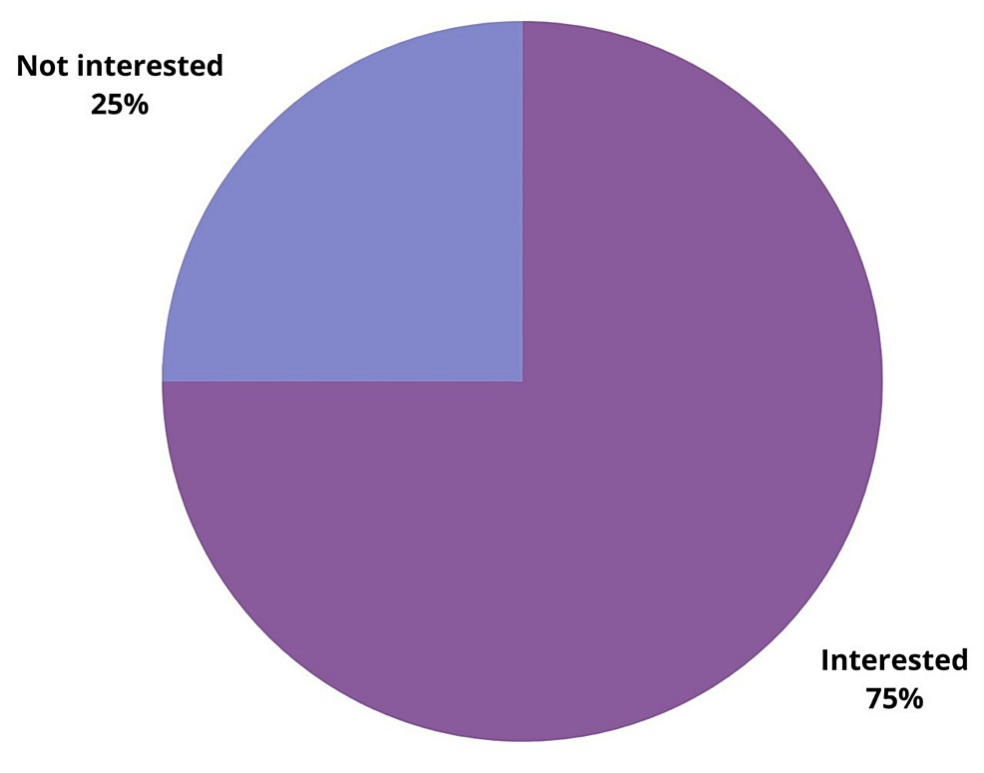
Participants who are interested in local fellowship training in Sudan

Of note, 75% of the participants were in favor of a local fellowship program (n=45) (Figure 4).

Differences between genders in terms of subspecialty choice were overt in this study; 52.2% of male trainees (n=12) selected "interventional radiology" as one of their choices, in contrast to 8.1% of females (n=3). On the other hand, while 32.4% of females (n=12) selected "women’s/mammogram imaging" as one of their choices, not a single male participant selected this subspecialty (Table 2).

**Table 2:** Gender variation with regard to the choice of subspecialty GI/GU: gastrointestinal/genitourinary; MRI: magnetic resonance imaging

Fellowship plan	Response count and %			
	Female, n	% (of 37)	Male, n	% (of 23)
Pediatrics	9	24.3%	4	17.4%
Body imaging, GI/GU imaging	11	29.7%	5	21.7%
Interventional radiology	3	8.1%	12	52.2%
Women's/mammogram imaging	12	32.4%	0	0.0%
Musculoskeletal imaging	6	16.2%	4	17.4%
Neuroradiology	12	32.4%	8	34.8%
Nuclear medicine	0	0.0%	1	4.3%
Cardiothoracic, thoracic, or cardiac imaging	1	2.7%	3	13.0%
MRI	2	5.4%	3	13.0%
Emergency radiology	1	2.7%	1	4.3%
Neurointerventional	2	5.4%	2	8.7%

A statistically significant association was found between gender and the choice of subspecialty in interventional radiology and women’s/mammogram imaging (p<0.001 and p=0.002, respectively). Also, a significant association was found between the fellowship choice and the decision-making time for interventional radiology, women's/mammogram imaging, and musculoskeletal imaging (Table 3). However, no significant association was found between the current year of training and the choice of subspecialty.

**Table 3:** Association of the choice of radiology subspecialty with demographics and the time of decision-making

Variables		Pediatrics	Body imaging, GI/GU imaging	Interventional radiology	Women's/mammogram imaging	Musculoskeletal imaging	Neuroradiology	Nuclear medicine	Cardiothoracic, thoracic, or cardiac imaging	MRI	Emergency radiology	Neurointerventional
Gender	Female	9	11	3	12	6	12	0	1	2	1	2
	Male	4	5	12	0	4	8	1	3	3	1	2
	P-value	0.526	0.496	<0.001	0.002	0.905	0.851	0.201	0.118	0.298	0.730	0.619
Current year of training	R2	3	4	6	6	4	3	1	0	3	0	2
	R3	5	5	6	2	2	9	0	2	1	2	1
	R4	5	7	3	4	4	8	0	2	1	0	1
	P-value	0.663	0.461	0.530	0.259	0.550	0.103	0.362	0.340	0.417	0.146	0.763
When did you decide to pursue subspecialty training?	After residency	7	11	5	12	10	15	0	3	5	1	1
	Before residency	3	3	9	0	0	3	1	1	0	1	3
	No plans to pursue	3	2	1	0	0	2	0	0	0	0	0
	P-value	0.469	0.930	<0.001	0.018	0.040	0.515	0.159	0.719	0.230	0.573	0.027

## Discussion

Our research examined the preference of radiology trainees with respect to different subspecialties and the factors affecting them. This study was conducted among 74 trainees, of which 60 responded, accounting for 66.7% (60/90) of the radiology trainees currently enrolled under SMSB, the body responsible for specialty training in Sudan. There were no first-year trainees at the time of this study because no radiology training selection exam had been conducted in Sudan since 2019.

The most popular subspecialties in our study were as follows: neuroradiology (33.3%), body imaging (26.7%), and interventional radiology (25%); this finding corresponds with the results of a study done in Saudi Arabia [10]. These results are also consistent with research done in UK and Singapore, where body imaging and neuroradiology were listed among the top three chosen subspecialties [11,12]. In addition, in West Africa, interventional radiology and neuroradiology were found to be trainees' top choices [13]. Although interventional radiology is among the most popular subspecialties chosen for a fellowship in our study, unfortunately, there is no training program available for this field in Sudan, and only four interventional radiologists are currently working in the country, all of whom gained their qualifications abroad [9].

Our analysis found that nuclear medicine (1.7%) and emergency radiology (3.3%) were among the least popular subspecialties. These results differ from those of prior research, where the unpopular subspecialties were pediatric imaging and oncology imaging [10-14]. However, our findings align with a study in the Netherlands, where only 8% of the trainees chose nuclear medicine as a subspecialty [15]. This low percentage may be due to the high stress and increased radiation exposure in nuclear medicine [16].

Of note, 21.7% of our participants indicated that they decided on pursuing a subspecialty before residency. This significant proportion is consistent with a study done in the US, where 25% of the participants made up their minds about fellowship plans before starting their residency [17]. In our research, choosing interventional radiology as a subspecialty was significantly associated with deciding upon subspecialization before starting radiology training (p<0.001). Many individuals who made their decision early may belong to this group. A study that examined the change of choices among doctors who select to pursue interventional radiology reported that this often changes after exposure to the field later on during training [18].

The top influencing factors in the selection of a subspecialty for our trainees included "strong personal interest," "lifestyle," and "area of strong personal knowledge," followed by "enjoyable rotation during residency" and "job market." The choices "selection by exclusion," "geographical limitation," "strengthening an area of weakness," and "influence of a role model" were among the minor prevalent factors in our study. These findings related to the most and least popular choices are somewhat similar to those in research done in Saudi Arabia, the UK, Singapore, and the US [10-12,17].

The majority of the trainees in our study (61.7%) were females, in contrast to research done in Saudi Arabia, West Africa, and the US, where female participants made up merely 35.2%, 27.4%, and 26.4%, respectively [10,13,19]. Women’s/mammogram imaging ranked fourth among the subspecialties chosen by 20% of the participants, all of whom were females. This observation regarding the exclusive choice of this subspecialty by females was also reported in the study done in Saudi Arabia [10].

There was a correlation between the time of deciding upon pursuing a subspecialty (after starting residency) and choosing mammography or musculoskeletal imaging as a subspecialty (p=0.018 and 0.040, respectively). This might be related to the trainees' exposure to the field during residency. Statistically significant differences were seen between genders regarding subspecialty choices in women’s imaging (p=0.02) and interventional radiology (p<0.01), which is consistent with the research in Saudi Arabia and Singapore [10,12]. This could be attributed to the fact that female doctors are more likely to pursue a career in women’s health [20]. The issue of male predominance in the field of interventional radiology was discussed in a study in Australia and New Zealand, concluding that the matter is complex and multifactorial, and the reasons given were related to lifestyle/on-call factors, time spent wearing lead gowns, family commitments, and radiation exposure [21].

More than 95% of our participants were located in Khartoum due to the private sector's dominance in training facilities and their well-equipped radiology departments [18]. Only 13.3% of our participants expressed a lack of interest in subspecialty training, among which 75% attributed it to the lack of fellowship training in Sudan. The most common factors preventing trainees from opting for fellowship were "financial restriction" (55%) and "family obligation" (28.3%); 75% were interested in joining a local program for fellowship training in the future in Sudan.

Recommendations

Radiologists from Sudan interested in subspecializing usually travel abroad for training; and once they find better prospects, many of them may not return. Instituting programs for subspecialization locally could mitigate the attrition of radiologists in Sudan. When designing subspecialty training programs in Sudan, stakeholders should rely on knowledge of influential factors and understanding of subspecialty decision trends as a reference point.

Limitations

This study has a few limitations. Primarily, this study used a convenient sampling technique, and hence its findings should be generalized with caution. Additionally, some of the correlations between the investigated parameters and the selection of subspecialties may have been obscured by the relatively small sample size.

## Conclusions

The trainees who participated in our study comprised 67.7% of the current radiology residents in Sudan. This study identified the popular subspecialty choices and the factors affecting the pursuit of subspecialty training among Sudanese radiology trainees. In addition, gender-based variations in selecting a fellowship were addressed. Based on our findings, despite the lack of availability of a subspecialization program in Sudan, almost two-thirds of the current radiology trainees want to pursue a fellowship after residency.
